# Histopathological dataset and demographic details of synovial tissues from patients with end-stage osteoarthritis, soft tissue and traumatic injuries of the knee

**DOI:** 10.1016/j.dib.2022.108082

**Published:** 2022-03-21

**Authors:** Juliana Jamal, Margaret M. Roebuck, Amanda Wood, Alasdair Santini, George Bou-Gharios, Simon P. Frostick, Pooi-Fong Wong

**Affiliations:** a Department of Musculoskeletal & Ageing Science, Institute of Life Course & Medical Sciences, University of Liverpool, Liverpool L7 8TX, UK; b Liverpool University Hospitals NHS Foundation Trust, Prescot Street, Liverpool L7 8XP, UK; c Department of Pharmacology, Faculty of Medicine, Universiti Malaya, Kuala Lumpur 50603, Malaysia; d Department of Molecular and Clinical Cancer Medicine, Institute of Translational Medicine, University of Liverpool, Liverpool L3 9TA, UK; e Faculty of Health and Life Science, The University of Liverpool, University of Liverpool, Liverpool L7 8TX, UK

**Keywords:** Cartilage debris, Cartilage inclusion, Inflammatory infiltrate, Macrophage, Peri-vascular, Peri-cartilage, Synovitis, Synovium

## Abstract

Degradation of articular cartilage is the defining feature of end-stage osteoarthritis (OA) with osteophytes, subchondral sclerosis, malalignment and joint space narrowing being additional indicators of advanced disease. Obesity, older age and female gender are OA risk factors. Differing degrees of synovitis are observed in OA, soft tissue and traumatic injuries of the knee. The synovium is also subject to systemic, enhanced lipids and inflammatory mediators characteristic of obesity. Synovial cellular composition changes specific to OA and associated with its handling of cartilage debris are unclear. Triangulation of data from three knee pathologies was used to highlight findings pertaining to OA compared to non-OA. OA patient data was compared to non-OA from knee ligament and tibial frature patients at surgery. Knee pathology, gender and BMI informed patient identification. Once consented, patient inclusion and characterisation utilised data from clinical assessments, blood tests, function scores, and radiological imaging, scores and intraoperative assessment. Intra-operative synovial tissues from the same site and processed identically underpins in-depth analyses and comparisons of histopathological images from these different knee pathologies. This supports the identification of distinct changes in the cellular composition of the knee synovium characteristic of OA. This data underpins a better understanding of OA pathogenesis and disease progression vital for the design of targeted therapeutics. The tissue and cell data include detailed results from the semi-quantitative synovitis score established by Krenn and observational data for morphological features such as cartilage debris inclusion, inflammatory cells aggregate and infiltration. This histopathological data is presented in the context of detailed clinical and functional information. This data and the holistic study design can be used as a foundation for the multifactorial collection and analysis of clinical data from OA patients, OA severity measures, tissue immuno-histology and synovial inflammation analysis to underpin the details and comparisons needed in further studies into OA and its treatment globally.

## Specifications Table

**Table:** 

Subject	Health and medical sciences (Orthopaedics, Sports Medicine and Rehabilitation)
Specific subject area	Contrasting histopathology of knee synovial tissues from osteoarthritic total knee replacement and non-osteoarthritic soft tissue and trauma injury patients
Type of data	Tables Images
How the data were acquired	Demographic and clinical parameters from consenting patients at the time of surgery with tissue collection Leica Aperio CS Slide Scanner for scanning IHC-stained slides up to 20X magnification
Data format	*Raw patient demographic information (xlsx)* *Analysed histological scores (xlsx)* *Raw digitally captures images (tif)*
Description of data collection	Knee patients requiring surgery provided demographic, health and functional score data: 16 endstage primary osteoarthritis, and 17 with no clinical or radiological evidence of arthritis, 9 ligament tears and 8 tibial plateau fractures. Rheumatoid arthritis (RA) and infection were excluded. Formalin fixed H&E-stained digitized medial gutter synovium 20x microimages were used for Krenn synovitis scoring and assessment of cartilage debris and inflammatory cells infiltration.
Data source location	Department of Molecular and Clinical Cancer Medicine, Institute of Translational Medicine, University of Liverpool, Liverpool L3 9TA, United Kingdom
Data accessibility	*Raw data is with the article. Images are available via Mendeley Data.* https://data.mendeley.com/datasets/cz3xt8mbpn/1

## Value of the Data

• Osteoarthritis is a chronic, painful, debilitating pathology impacting billions of people worldwide and costing billions in lost earnings and treatment [1,2]. While cartilage degradation is well understood the pathophysiological development of OA is unclear and treatments to slow progression entirely missing [3]. Animal data is interesting, but human studies are limited and difficult, well conducted studies very rare.
• Critical to the value of these data is that the tissues were collected and studied with their detailed provenance known. Obtaining normal healthy synovium from individuals is ethically unsound preventing the ideal comparison of OA synovium with healthy synovium. The best alternative is to compare tissues from well characterised distinctive groups of patients. A detailed review both pre- and intra-operatively, allowed appropriate samples to be evaluated.
• The data is comprehensive at several levels, patient, knee joint, tissue and histopathological including standard knee scores and synovitis with detailed observations of cartilage debris inclusion and inflammatory cells infiltration.
• The data and histological images presented underpin comparison of synovial tissues from end-stage osteoarthritis with soft tissue injury and knee trauma injury with no osteoarthritic features. This has revealed specific histological changes in the synovial tissues of end-stage osteoarthritis.
• This data contributes to a better understanding of OA pathogenesis and supports multifactorial analysis of clinical data, osteoarthritis severity measures, tissue immune histology and synovial inflammation analysis.
• As OA and its treatment vary between nations and health systems, this data and the study design can be used to clarify the details needed in further studies into OA

## 1 Data Description

Table 1 details the individual patient's clinical demography including patient's pathology, gender, age, body mass index (BMI), duration of symptoms, blood pressure, leukocytes count, C-reactive protein (CRP), S100a8a9 protein level, American Society of Anesthesiology (ASA) physical status classification grade, cumulative illness rating scale, The Western Ontario and McMaster Universities Osteoarthritis Index (WOMAC) score, 12-item Short Form Survey (SF-12) physical component score and SF-12 mental component score [4].

**Table 1:** Individual patient's clinical data demography.

Sample i.d.	Pathology	Gender	Age (years)	BMI (kg/m^2^)	Duration of symptoms (years)	BP (mmHg)	WCC x10^9^/L	CRP (mg/l)	S100a8a9	ASA grade	CIRS	WOMAC score	SF-12 Ph	SF-12 M
16	OA	M	72.06	35.93	10.00	150/70	8.4	1	6.05	2	7	59	34.80	50.40
29	OA	F	60.05	28.59	9.00	157/79	10.3	1	5.26	1	3	72	25.50	54.40
31	OA	M	69.62	32.19	27.00	160/90	6.9	1	6.78	2	7	66	21.00	57.20
35	OA	M	53.63	32.55	3.00	120/80	7.2	1	5.42	2	5	82	23.90	56.40
36	OA	F	57.83	34.22	20.00	125/70	6.6	1	6.37	1	3	66	36.20	26.50
43	OA	F	63.67	29.02	5.00	135/83	7.2	1	6.64	2	9	69	13.20	68.30
44	OA	M	71.73	32.70	5.00	140/80	6.1	1	5.07	2	8	36	34.90	68.00
48	OA	F	67.44	24.27	10.00	141/90	7.0	1	6.37	2	6	82	16.90	62.80
70	OA	M	58.75	25.61	1.00	135/82	6.4	8	5.25	2	10	66	30.99	45.30
75	OA	M	57.48	26.87	7.00	145/89	6.0	1	5.88	2	4	77	33.55	52.03
78	OA	F	59.95	36.88	3.00	150/90	4.1	1	5.23	2	6	71	32.83	45.48
89	OA	F	73.90	25.62	10.00	150/80	5.7	1	4.05	2	6	54	34.96	68.01
90	OA	M	55.40	28.73	20.00	132/85	7.3	1	5.22	2	4	96	19.15	60.41
98	OA	F	56.00	37.48	2.00	99/63	6.8	19	9.57	2	9	72	29.18	42.75
99	OA	M	76.01	26.43	10.00	139/79	6.7	1	5.54	2	4	45	25.13	66.02
169	OA	F	54.66	33.56	3.00	149/88	4.4	1	-	2	9	65	26.71	40.03
18	Scope	F	33.96	26.30	0.50	110/80	6.1	1	4.52	1	2	6	44.00	66.70
59	Scope	F	26.32	21.36	1.00	100/64	6.9	1	5.47	1	1	1	46.44	60.73
60	Scope	M	25.97	19.88	1.00	110/60	6.1	1	4.27	1	4	6	39.19	63.50
61	Scope	M	25.68	24.16	1.50	120/80	6.4	1	5.68	1	2	1	51.06	59.29
81	Scope	M	28.71	25.53	1.00	128/75	5.4	1	6.02	1	1	11	33.86	68.66
88	Scope	M	29.16	25.25	1.00	120/85	14.4	1	4.50	1	1	-	-	-
91	Scope	F	27.17	22.25	0.50	132/78	7.1	1	3.71	1	2	9	46.70	64.96
120	Scope	F	33.03	23.97	0.50	115/72	5.4	1	10.17	1	1	22	39.26	63.85
155	Scope	F	25.25	24.06	1.50	120/70	8.8	1	-	1	1	1	52.76	58.52
46	Trauma	M	37.33	24.19	0.02	123/79	7.6	61	4.80	1	3	-	-	-
54	Trauma	F	54.33	27.00	0.04	114/80	6.8	18	5.21	2	12	-	-	-
55	Trauma	M	46.00	39.20	0.04	111/71	12.2	-	-	1	6	-	-	-
58	Trauma	F	63.00	30.60	0.02	146/81	11.7	-	5.44	2	7	-	-	-
62	Trauma	M	29.27	21.40	0.04	150/81	7.2	-	-	2	6	-	60.10	34.77
63	Trauma	F	34.82	48.43	0.04	139/90	9.2	15	3.43	2	5	-	61.19	41.46
84	Trauma	F	24.42	19.79	0.02	120/78	5.4	6	2.30	1	0	-	46.31	40.36
95	Trauma	M	19.34	23.44	0.02	-	6.1	19	6.41	1	1	-	26.28	66.34

**Clinical range guidelines recommended by the NHS UK:**

BMI: Body mass index (kg/m^2^): normal,18≤BMI<25; overweight, 25≤BMI<30; obesity, BMI≥30

BP: Systolic and diastolic blood pressure (mmHg): ideal BP, between 90/60 – 120/80; high BP>140/90; at risk of developing high BP, between 120/80 – 140/90

WCC: White blood cells counts (x10^9^/L): normal, 3.8–10.8

CRP: C-reactive protein (mg/l), normal range: <5

S100a8a9: Constitutively expressed in neutrophils and monocytes, a predictive biomarker for diagnosis of inflammation-associated diseases.

ASA: Physical status classification grade >1; patients with systemic disease, mild or severe

CIRS: Cumulative illness rating scale total: morbidity count, which indicates the number of diseases on which the patients scored a severity of 2 or higher.

WOMAC: Western Ontario and McMaster Universities Osteoarthritis Index

SF-12 Ph: The 12-item Short Form Survey, a physical component score

SF-12 M: The 12-item Short Form Survey,  a mental component score

Table 2 summarises individual patient's synovial tissue histology analysis which include pain score, a subcomponent of WOMAC score; synovitis score, a sum of synovial lining hyperplasia grade, density of the resident score grade and inflammatory infiltrate grade. The total number of image grids captured for each patient's tissue is shown [4].

**Table 2:** Individual patient's synovial tissue histology analysis.

Sample i.d.	No of image grids	Pain Score	Synovitis grade	Synovial lining hyperplasia	Density of the resident cells	Inflammatory infiltrate	Presence of cartilage inclusion	Diffuse infiltrate	Peri-vascular focal infiltrate	Peri-cartilage
16	12	7	5	2	1	2	no	yes	no	no
29	15	10	7	3	2	2	yes	no	no	no
31	7	9	-	-	-	-	no	yes	no	no
35	11	5	8	2	3	3	yes	yes	yes	yes
36	0	10	-	-	-	-	ND	ND	ND	ND
43	26	8	8	3	2	3	yes	yes	yes	yes
44	21	5	6	2	2	2	no	yes	yes	no
48	22	10	8	3	3	2	yes	yes	yes	yes
70	8	7	7	3	2	2	yes	yes	yes	no
75	25	9	8	3	2	3	yes	yes	yes	no
78	25	10	7	2	2	3	yes	yes	yes	no
89	14	6	8	3	2	3	yes	yes	yes	yes
90	16	10	8	3	2	3	yes	yes	yes	yes
98	40	8	9	3	3	3	yes	yes	yes	no
99	17	4	7	3	2	2	yes	yes	yes	yes
169	36	5	6	2	2	2	yes	yes	no	no
18	5	-	-	-	-	-	no	no	no	no
59	10	-	7	2	3	2	no	yes	no	no
60	16	-	5	1	2	2	no	yes	no	no
61	5	-	5	1	2	2	no	yes	no	no
81	5	-	6	2	2	2	no	yes	no	no
88	10	-	6	2	2	2	no	no	no	no
91	7	-	7	2	3	2	no	yes	no	no
120	4	-	5	1	2	2	no	no	no	no
155	9	-	5	2	2	1	no	yes	no	no
46	10	-	7	2	3	2	no	yes	no	no
54	4	-	9	3	3	3	no	yes	no	no
55	10	-	-	-	-	-	no	yes	no	no
58	14	-	5	1	2	2	no	yes	no	no
62	5	-	3	1	1	1	no	yes	no	no
63	4	-	6	1	3	2	no	yes	no	no
84	12	-	-	-	-	-	no	yes	no	no
95	5	-	8	2	3	3	no	yes	no	no

ND: Not determined

Sample i.d. 36 did not have any histological assessment results due to handling issues during tissue processing, hence were excluded from analysis.

Pain score: evaluated by WOMAC pain questionnaires during walking, using stairs, in bed, sitting or lying, and standing upright with a possible score range of 0-20. Higher scores indicate worse pain.

Synovitis grade: sum of all three morphological features score of synovial lining hyperplasia, density of the resident cells and inflammatory infiltrate score. Sum 0 to 1 indicates no synovitis, sum between 2 to 4 indicates low-grade synovitis and sum between 5 to 9 shows high-grade synovitis. The grading criteria of synovial lining hyperplasia, density of the resident cells and inflammatory infiltrate score are as described in Krenn *et al*., (2006).

For observational analysis of the presence of cartilage inclusion, diffuse inflammatory immune cells infiltration, peri-vascular focal inflammatory immune cells infiltrate, and peri-cartilage focal inflammatory immune cells infiltrate was stated as a yes or no nominal response.

Table 3 lists individual OA patient's radiology results for OA severity assessment that include scoring of various components such as extensivity of osteophyte, joint-space loss, sub-chondral sclerosis, varus joint deformity, Kellgren-Lawrence and intraoperative observation notes [4].

**Table 3:** Radiologic findings of knee OA severity assessment.

	Osteophytes		Joint Space Loss		Subchondral Sclerosis		Deformity		K-L	
Sample i.d.	Location	Grade	Location	Grade	Location	Grade	Description	Grade	Grade	Intraoperative observation notes
29	Medial tibia; superior & inferior patella; lateral anterior & posterior femur	1	Absent on medial side	2	Medial tibial plateau	2	Moderate varus	2	3	Grade IV changes medially, Grade III changes patellofemoral, Grade II changes laterally.
43	Medial femur, tibia, patella	1	Decreased on medial side	1	No	0	Mild varus	1	2	Severe medial OA
48	Lateral tibial plateau, medial femoral condyle; patella	2	Severely decreased on lateral side	2	Lateral tibial condyle	1	Mild valgus	1	3	Grade IV changes medially
70	Medial femoral condyle	1	Moderate decrease on medial side	1	No	0	Mild varus	1	3	PFJ Abnormalities: Generalised degeneration
75	Extensive distal femur & patella	1	Decreased on medial side	1	No	0	Mild varus	1	3	Proximal tibial deformity
89	Distal femur; patella	2	Decreased on medial side	1	No	0	Mild varus	1	3	Marked OA
90	Mild femur & patella	1	Virtually absent medial compartments	2	Medial tibial plateau	1	Mild varus	1	4	Marked OA
99	Extensive distal femur & patella	3	Absent on both medial & lateral compartments but severe collapse on medial side	3	Medial tibial plateau	1	Severe varus	3	4	Marked OA. Varus. Severely deformed FFD. Deficient posterio-medial tibial condyle
16	Tibia, femur & patella but less on tibia	2	Absent medial side	2	Minimal	1	Moderate varus	2	4	Femur: Grade IV both condyles
31	Extensive	2	Virtually absent medial compartment	2	Minimal	1	Mild varus	1	4	Moderate synovitis Tibial plateau:Grade IV medial & Grade II lateral Femoral condyles: Grade IV medial & Grade II lateral Patella: Grade III OA Medial facet, Grade II OA Lateral facet
35	Mild femur & patella	1	Markedly reduced on medial side	2	No	0	Mild varus	1	3	Grade IV changes medially Grade II changes patellofemoral Grade II changes laterally.
36	Mild femur & tibia	1	Difficult because of malalignment; some loss	1	Medial tibial plateau	1	Moderate varus	2	3	Grade I changes laterally malalignment of femur on tibia; possible ligament instability
44	Femur, tibia, patella	2	Virtually complete loss on medial compartment	2	Medial tibial plateau	1	Mild varus	1	4	Grade IV changes laterally Grade IV changes patellofemoral joint Grade IV changes medially
78	Mild femur, tibia, patella	1	Significant decrease on medial side	1	No	0	Mild varus	1	3	Tri-compartment osteoarthritis with severe arthritis of the knee medial side
98	Minimal	1	Slight decrease medial compartment	1	No	0	Possibly very Mild varus	1	2	General: Significant synovitis, arthritic changes, serous fluid
169	Mild femur & tibia, patella	1	Moderate to severe loss lateral compartment	2	No	0	Moderate valgus	2	3	PFJ grade IV Medial compartment, grade IV Lateral compartment, and grade IV Possible history of Lateral tibial plateau injury

Individual radiographic features in knee OA patients include grading of osteophytes, joint space loss or narrowing, subchondral sclerosis, varus and valgus deformity and K-L (Kellgren-Lawrence) score.

PFJ: Patello femoral joint

FFD: Fixed flexion deformity

**Fig. 1:**
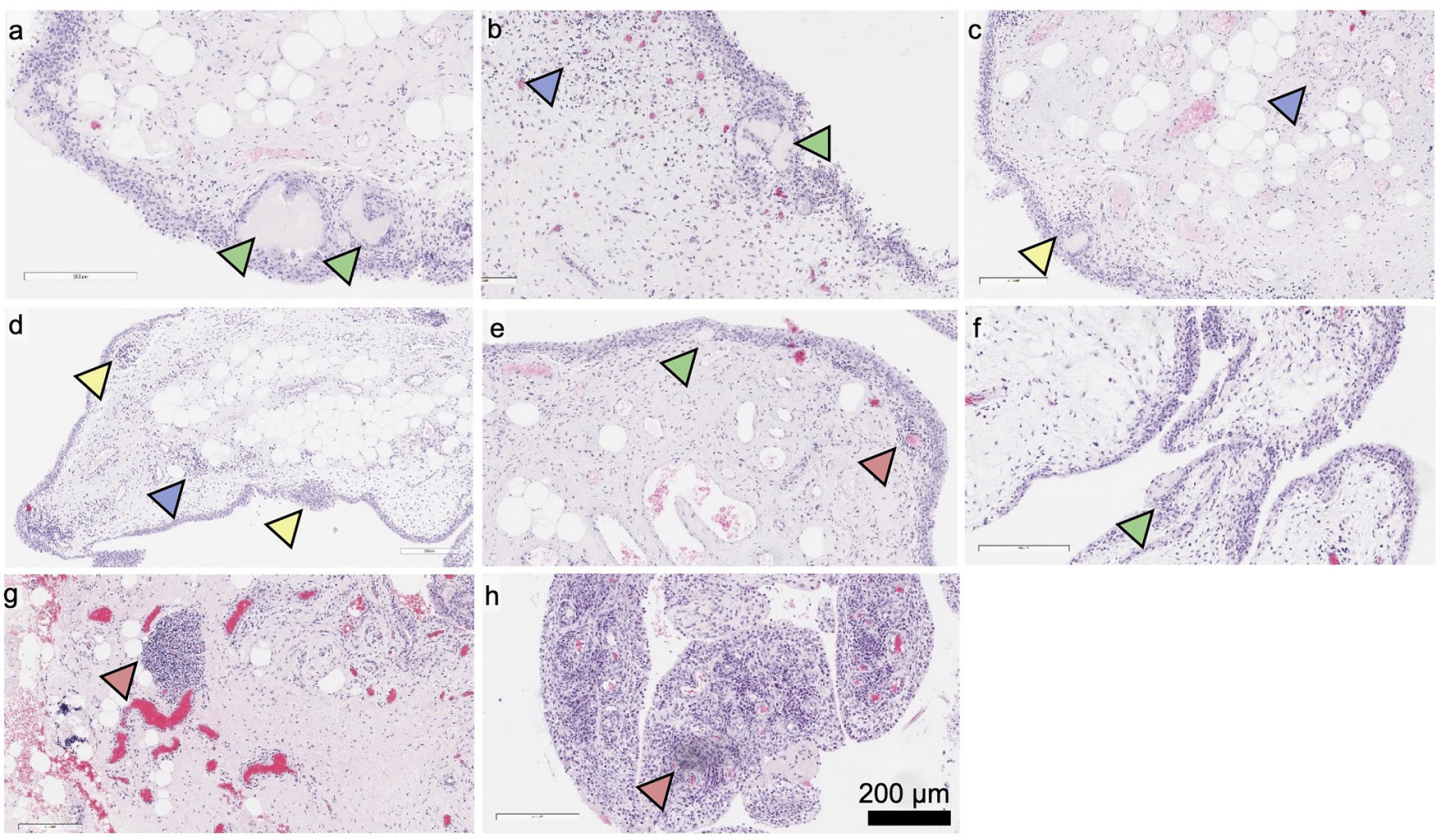

Figure 1 shows representative images of H&E-stained OA-synovial tissues (Figure 1a-h). Observation of cartilage debris inclusion is indicated by green arrowheads. Focal inflammatory cells infiltrate at the synovial subintima layer is shown by blue arrowheads. Focal inflammatory cells surrounding the cartilage debris inclusion, termed as peri-cartilage, and peri-vascular focal inflammatory cells infiltrate are indicated by yellow and red arrowheads, respectively.

The repository contains 32 individual patient folders, except for sample i.d. 36 which was excluded due to technical issues during tissue processing. Individual folder comprised of haematoxylin and eosin (H&E)-stained synovial tissue section images. Each H&E folder consists of sequential image grids, a full tissue image labeled as HE_sample id_full and image grids trail map labeled as HE_sample id_map.

## 2 Experimental Design, Materials and Methods

### 2.1 Experimental design

A triangulation of standardised factors and data for three distinct cohorts of knee pathologies, one group with osteoarthritis and two without osteoarthritis but with different knee insults, ligament tears and tibial plateau fractures allowed the analysis of OA specific changes. Known risk factors for OA were incorporated into the cohort definition criteria, gender was balanced, BMI distribution selected between, a healthy or an obese BMI. Tissues and data from well characterised patients were analysed. Age remained a confounding factor.

### 2.2 Patients

All tissue samples utilised in this study were obtained during knee surgery at the Liverpool University Hospitals NHS Foundation Trust, Liverpool, United Kingdom. Following patient informed consent (Medical ethics approval reference: 15/NW/0661), synovial tissue samples and associated clinical data were donated to the Liverpool Musculoskeletal Biobank (LMB). Synovial biopsied were collected from 33 patients, 16 end-stage OA, 9 scope and 8 trauma-injury patients. Arthroplasty patients (OATKR), were selected to balance gender (M:F) and BMI classification; 4M:BMI<30, 4M:BMI>30, 4F:BMI<30 and 4F:BMI>30. For OA-TKR, only patients with presumed primary OA were included, patients with post-traumatic or rheumatoid arthritis (RA) were excluded. Other exclusion criteria include patients who were unable or unwilling to provide written informed consent, patients presented with lower limb gangrene or peripheral vascular disease, patients with history of hepatic or renal impairment or dialysis, patients known to be HIV positive of patients of high risk of this conditions such as intravenous drug users both past and present, patients currently receiving or has received radiation or chemotherapy within the last three months and patients under current use of systemic cortisone. Relevant individual patient's clinical data including the patient's age, gender, duration of knee pain symptoms, BMI, blood pressure, white blood cell counts, medical history, CRP inflammation blood tests, Kellgren Lawrence and pain score as the indicators of OA severity were recorded where applicable. Intra-operative synovial tissue was biopsied at the medial gutter. Following sample collections, each tissue sample was linked-anonymised using the patient's unique study number (sample i.d.). Upon collection, fresh synovium tissue was placed in a container with 10% formalin for routine histological formalin-fixed paraffin embedded tissue processing.

### 2.3 Immunohistochemical labelling

Formalin fixed paraffin embedded synovial biopsied were processed for standard haematoxylin and eosin (H&E) staining by the Liverpool Bio-Innovation Hub (LBIH) Biobank, Liverpool, UK. High-resolution images of H&E-stained tissues were acquired and digitalized at 20x using Aperio CS2 Digital Pathology Scanner (Leica Biosystems, USA). Microscopic images of H&E tissue sections were captured at 10x using Aperio ImageScope v12.3 (Leica Biosystems, USA).

### 2.4 Synovitis scoring

Using the repository images of primary OA synovial tissue specimens, all areas of each biopsy section were examined, and histological features of synovitis were scored independently by two assessors on three major synovitis components of synovial lining hyperplasia, activation of resident cells (stroma) and inflammatory infiltrate, with some modifications adapted from Krenn synovitis scoring. The synovitis scoring criteria is adapted from [5,^6^,7]. The original synovitis scoring proposed by Krenn *et al.* (2006) was introduced to assess synovial membrane histopathology to discriminate between the OA, post-traumatic arthritis, rheumatoid arthritis, psoriatic arthritis, reactive arthritis, and synovial tissues autopsies of patients without joint damage. There are no changes made to the total grading summary, whereby a total synovitis score of 0 or 1 indicates no synovitis, score of 2 to 4 indicates a low-grade synovitis and score of 5 to 9 marks a high-grade synovitis. Interscorer variability testing was performed using Cohen's Kappa statistical test.

### 2.5 Cartilage debris inclusion and inflammatory cells infiltration analysis

Digitised H&E images at 20x were examined for focal concentrations of cells and areas of acellularity. Areas of focal concentrations of cells were further examined for CD3, CD20, CD68 and von Willebrand factor (vWF) positivity to determine the type of inflammatory cell infiltration and vascularisation. Areas of acellularity were examined to exclude possible staining or cutting artifacts, residual fibrin or other possible blood product inclusion. Serial sections were aligned and the area of acellularity followed through the depth of the tissue fragment. The tissue locations with cartilage inclusions were examined for CD3, CD20, CD68 positivity. From these serial alignments a perivascular or peri-cartilage location could be ascribed to each inflammatory aggregates. Those tissues with no such aggregations were described as having a diffuse pattern of inflammatory cells.

## Ethics Statements

**Ethics approval**: North West - Liverpool Central REC reference: 15/NW/0661. This research has been conducted in accordance with The Code of Ethics of the World Medical Association (Declaration of Helsinki).

**Consent to participate:** All patients gave informed consent and donated samples and associated clinical data to the Liverpool Musculoskeletal Biobank (LMB; Management team – SPF biobank CI, AW).

## Funding

This work was supported by the Fundamental Research Grant Scheme of The Ministry of Higher Education Malaysia (FRGS/1/2018/SKK08/UM/02/26 (FP011-2018A)). JJ was a recipient of MyPhD Scholarship from the Ministry of Higher Education, Malaysia and University of Malaya Dual PhD Program.

## CRediT authorship contribution statement

**Juliana Jamal:** Methodology, Formal analysis, Investigation, Visualization, Writing – original draft. **Margaret M. Roebuck:** Conceptualization, Methodology, Formal analysis, Writing – review & editing. **Amanda Wood:** Resources. **Alasdair Santini:** Writing – review & editing. **George Bou-Gharios:** Conceptualization. **Simon P. Frostick:** Conceptualization, Funding acquisition. **Pooi-Fong Wong:** Conceptualization, Funding acquisition, Writing – review & editing, Supervision.

## Declaration of Competing Interest

The authors declare that they have no known competing financial interests or personal relationships that could have appeared to influence the work reported in this paper.

The authors declare the following financial interests/personal relationships which may be considered as potential competing interests.

## Data Availability

Synovial tissues histology from patients with end-stage osteoarthritis, soft tissue and traumatic injuries of the knee (Original data) (Mendeley Data).

## References

[1] Mobasheri A., Saarakkala S., Finnilä M., Karsdal M.A., Bay-Jensen A.-C., van Spil W.E. (2019). Recent advances in understanding the phenotypes of osteoarthritis. F1000Research.

[2] Mobasheri A., Batt M. (2016). An update on the pathophysiology of osteoarthritis. Ann. Phys. Rehabil. Med..

[3] Mathiessen A., Conaghan P.G. (2017). Synovitis in osteoarthritis: current understanding with therapeutic implications. Arthritis Res. therapy.

[4] Jamal J., Roebuck M., Wood A., Santini A., Bou-Gharios G., Wong P.-F. (2022). Synovial tissues histology from patients with end-stage osteoarthritis, soft tissue and traumatic injuries of the knee. Mendeley Data.

[5] Krenn V., Morawietz L., Burmester G.R., Kinne R.W., Mueller-Ladner U., Muller B., Haupl T. (2006). Synovitis score: discrimination between chronic low-grade and high-grade synovitis. Histopathology.

[6] Krenn V., Morawietz L., Haupl T., Neidel J., Petersen I., Konig A. (2002). Grading of chronic synovitis–a histopathological grading system for molecular and diagnostic pathology. Pathol. Res. Pract..

[7] Najm A., le Goff B., Venet G., Garraud T., Amiaud J., Biha N., Charrier C., Touchais S., Crenn V., Blanchard F., Krenn V. (2019). IMSYC immunologic synovitis score: A new score for synovial membrane characterization in inflammatory and non-inflammatory arthritis. Joint Bone Spine.

